# Intravital imaging of immune responses in intestinal inflammation

**DOI:** 10.1186/s41232-023-00262-w

**Published:** 2023-02-03

**Authors:** Masaki Honda, Masashi Kadohisa, Daiki Yoshii, Yoshihiro Komohara, Taizo Hibi

**Affiliations:** 1 Department of Pediatric Surgery and Transplantation, Kumamoto University Graduate School of Medical Sciences, 1-1-1 Honjo, Chuo-Ku, Kumamoto, 860-8556 Japan; 2 Department of Diagnostic Pathology, Kumamoto University Hospital, Kumamoto, Japan; 3 Department of Cell Pathology, Kumamoto University Graduate School of Medical Sciences, Kumamoto, Japan

**Keywords:** Intravital imaging, Immune cells, Intestinal inflammation, Colorectal cancer, Tissue repair

## Abstract

To date, many kinds of immune cells have been identified, but their precise roles in intestinal immunity remain unclear. Understanding the in vivo behavior of these immune cells and their function in gastrointestinal inflammation, including colitis, inflammatory bowel disease, ischemia–reperfusion injury, and neutrophil extracellular traps, is critical for gastrointestinal research to proceed to the next step. Additionally, understanding the immune responses involved in gastrointestinal tumors and tissue repair is becoming increasingly important for the elucidation of disease mechanisms that have been unknown. In recent years, the application of intravital microscopy in gastrointestinal research has provided novel insights into the mechanisms of intestine-specific events including innate and adaptive immunities. In this review, we focus on the emerging role of intravital imaging in gastrointestinal research and describe how to observe the intestines and immune cells using intravital microscopy. Additionally, we outline novel findings obtained by this new technique.

## Background

The gastrointestinal tract is a very complex integrated organ that includes a prominent intestinal immune system and plays an important role in life support, including digestion and absorption, while coexisting with the gut microbiome. There is no question about the importance of research into intestinal inflammation, but most findings to date have been based on static evaluation of histological sections, flow cytometry, or an alternative index such as the myeloperoxidase activity. Because conventional research methods do not provide insights into the nature of cellular interactions in vivo during intestinal inflammation, more physiological in vivo, real-time mechanistic analysis has been desired. Recent progress of intravital microscopy (IVM) has enabled the visualization and quantification of immune cell recruitment in vivo [1]. It provides invaluable information about immune cell motion, proliferation, and death processes, as well as cell–cell interactions at the single-cell resolution in a number of organs and disease models. In recent years, IVM has played an emerging role in intestinal research, yielding many insights that were not possible by previous methods. Furthermore, with the development of various transgenic mice and fluorescent antibodies, the subdivision of immune cells that can be observed and the application of IVM are expanding.

This review describes how IVM is used to image the behavior of the gut and associated immune cells during steady-state or inflammatory conditions. We also provide an overview of the invaluable findings obtained by this novel technique with respect to intestinal inflammation, cancer, and tissue repair.

## Intravital imaging of the gastrointestinal tract

The development of microscopy techniques from simple light microscopy to confocal microscopy, including laser scanning and spinning disk, and two-photon laser scanning microscopy (TPLSM) has had a significant effect on studying immune responses in living organs using IVM [2, 3]. In a confocal microscope, light is focused to a point and emitted fluorescence passes through a pinhole before reaching the detector. A spinning disk consists of multiple pinholes on a rotating disk, which has the advantage of shortening the scanning process. However, the penetration depth of confocal microscopy is limited, making it difficult to image all layers of the intestinal wall. In TPLSM, a pulsed laser directs two excitation photons of approximately half the energy onto the sample. When these two, low-energy photons hit the fluorophore simultaneously, they are excited to the same level as one high-energy photon. This principle provides numerous advantages including high resolution, deep site imaging of at least 100 µm below the organ surface, less phototoxicity, and less photobleaching compared with conventional confocal microscopy. These advantages make it suitable for long-term imaging of pathophysiological changes in all layers of the intestinal tract. These properties of microscopies have been discussed elsewhere in detail [2–4], and we focus on intestinal imaging and the findings obtained by IVM in this review.

**Fig. 1:**
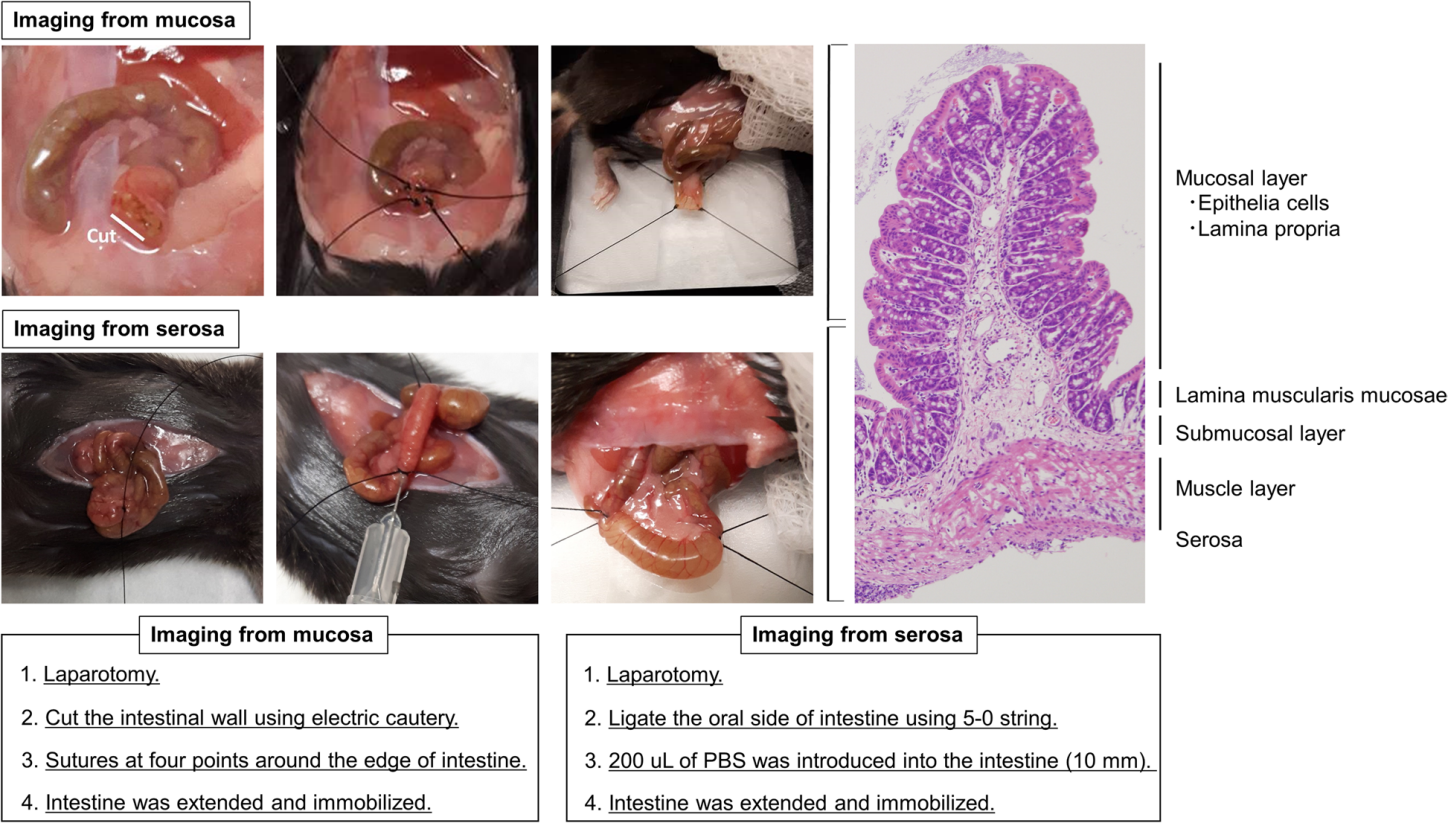
Preparation for intravital intestinal imaging using an inverted microscope. Representative intestinal fixation method for intravital intestinal imaging from the mucosa (upper row) or serosa (lower row) using an inverted microscope. Details of each method are described in the box below

**Fig. 2:**
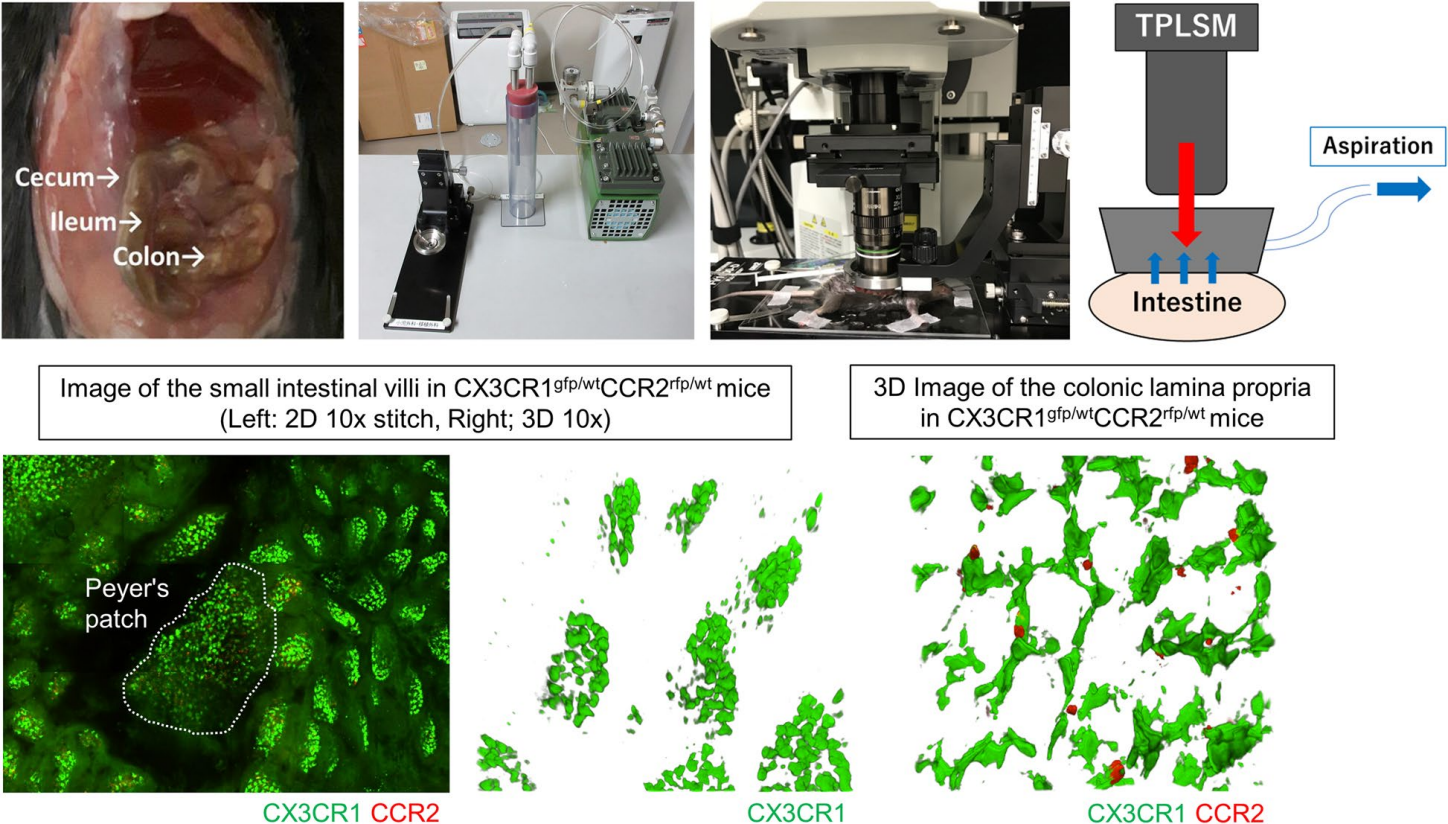
Preparation for intravital intestinal imaging using an upright microscope. The upper row shows a typical intestinal fixation method for in vivo intestinal imaging using an upright microscope. The intestine is fixed using a suction window. The lower row shows representative intestinal images in CX3CR1^GFP/+^CCR2^RFP/+^ mice

Difficulty in controlling intestinal peristalsis and flattening is a major issue when applying intravital imaging to the intestinal tract. To reduce motion artifacts and perform optimal imaging, it is necessary to fix organs using a suction window or glue, appropriate anesthesia to minimize motion artifact, administration of butylscopolamine, or expanding the intestinal wall [5–7]. Imaging preparation has been developed on the basis of certain conditions such as the target intestinal layer and whether the microscope is inverted or upright (Figs. 1 and 2). A confocal microscope has a limited capacity to image deep into the intestine, and therefore, the optimal images can be obtained by observing from the side of the object to be observed, whether it is the mucosa or the serosa. We believe that it is important to understand the aforementioned pros and cons of the confocal microscope and TPLSM and to select the microscope that provides the best image of the subject (i.e., intestinal layer, immune cells, disease model, etc.) to be observed. Notably, Rakhilin et al. reported an intravital colonic window using a ferromagnetic scaffold for chronic imaging [8]. Using this technique, they imaged fluorescently labeled Lgr5-positive stem cells, bacteria, and immune cells in the live murine colon. In oncological studies, a surgical orthotopic organoid transplantation approach has been used to visualize colorectal cancer progression in vivo [9].

**Fig. 3:**
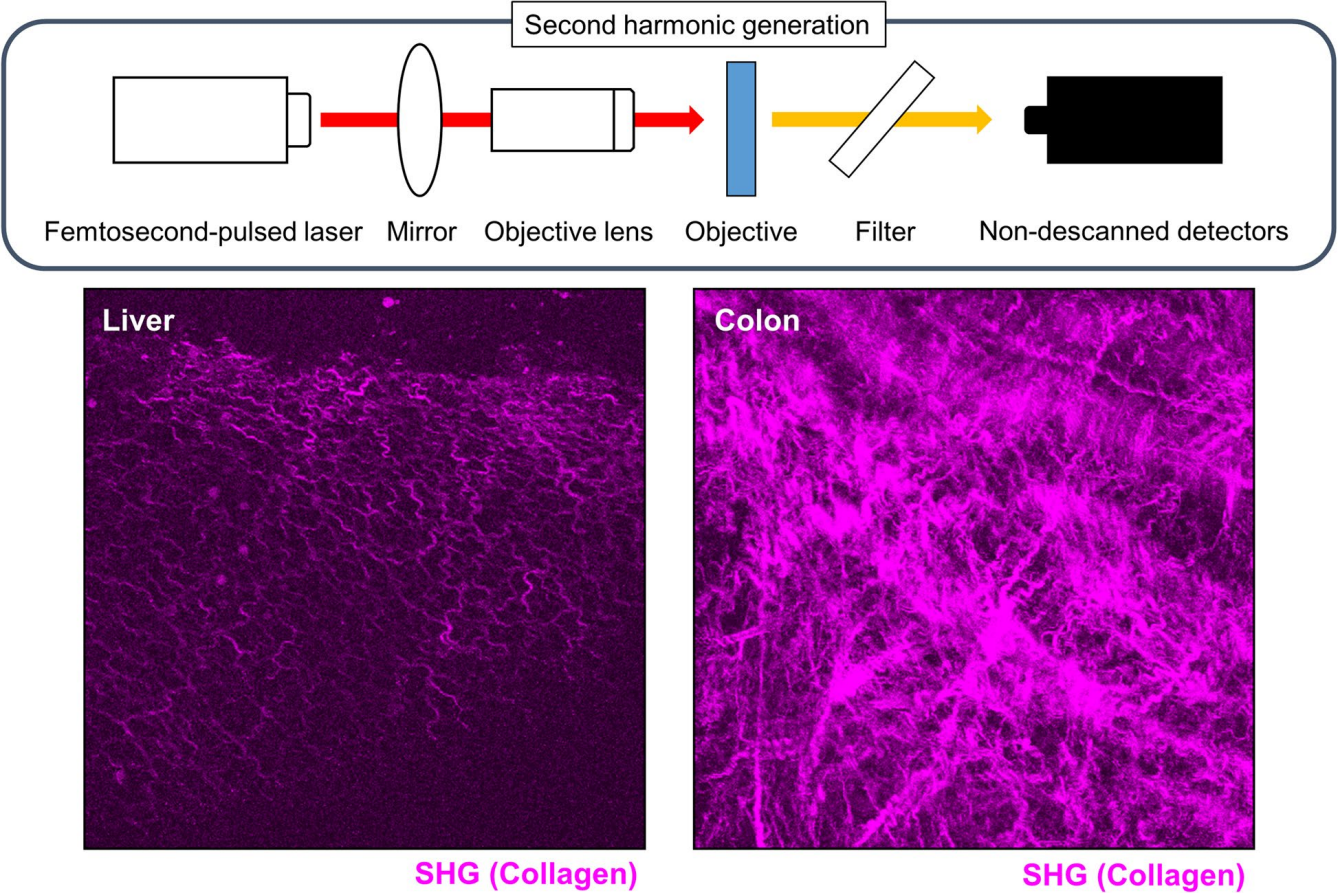
Imaging of the second harmonic generation using a two-photon laser scanning microscope. Representative images of second harmonic generation (SHG) in the liver (left) and colon (right) at the steady state obtained under a two-photon laser scanning microscope

Various fluorescent reporter mice are available for intestinal imaging using IVM. In fact, many new findings have been obtained by capturing the real-time movement of immune cells in the inflamed intestinal tract (Table 1). Specific fluorescent antibodies are similarly useful to identify immune cells and delineate intestinal structures such as blood vessels and lymphatic capillaries [10, 11]. As a novel technique for intravital imaging, mesenteric collecting lymphatic vessel cannulation has enabled in vivo lymph flow assessment [12]. Additionally, Orzekowsky-Schroeder et al. demonstrated the utility of TPLSM excited autofluorescence to differentiate the cell types in living intestines [13]. Intriguingly, metabolic labeling of gut anaerobic bacteria has enabled visualization of the anaerobic microbial niche by various methods, such as IVM and non-invasive whole body imaging, which can be used to observe microbial colonization and host–microbe interactions in real time [14]. Moreover, when an ultrashort pulsed laser beam in the order of femtoseconds is irradiated onto an asymmetric material such as a crystal, light with half the wavelength or twice the frequency of the incident laser beam is emitted. In recent years, with the development of ultrashort pulse lasers such as TPLSM, they have also been used for biological imaging [15]. Second harmonic generation (SHG) is induced in vivo by collagen, myosin, and tubulin. In particular, collagen is abundant and generated with high efficiency. Therefore, it can be visualized specifically in living tissue by SHG imaging and observed without staining (Fig. 3). Maier et al. imaged layers within murine colorectums via SHG and showed that submucosa had the largest collagen fiber diameters, followed by serosa and muscle [16]. They also showed that collagen fibers aligned with muscle fibers in the two muscular layers. These findings would support to identify which of the layers we are observing during intestinal imaging. Moreover, SHG imaging is useful for observing how collagen is produced in response to intestinal inflammation, infection, and tumor development.

**Table 1:** Fluorescent-labeled mice for intravital imaging of immune cells in the intestine

Target gene/promotor	Fluorescence	Labeled cells	Disease model	Observed phenomena	Reference
LysM	eGFP	Neutrophils	Intestinal ischemia–reperfusion Celiac disease	Cell recruitment/extravasation Cell recruitment/accumulation	Hashimoto et al. [5] Lammers et al. [17]
CCR2	RFP	CCR2 + monocytes	DSS colitis	Changes of cellular phenotype	Honda et al. [18]
CX3CR1	GFP	CX3CR1 + monocytes/macrophages	DSS colitis Salmonella infection	Changes of cellular phenotype Phagocytic function	Honda et al. [18] Niess et al. [19]
CX3CR1 × CD11c	GFP × YFP	Dendritic cells	Salmonella infection	Phagocytic function	Farache et al. [20]
TCRδ	eGFP	TCRδ + intraepithelial lymphocytes	Indomethacin/LPS-induced intestinal leakage	Changes in cell movement Intercellular contacts	Sumida et al. [21] Hu et al. [22]
Foxp3	tdTomato	Regulatory T cells	Intestinal inflammation	Changes in cell movement	Sujino et al. [23]
CD2 × IL13	GFP × tdTomato	Group 2 innate lymphoid cells	Helminth infection	Intercellular contacts	Lok et al. [24]
Rorc × IL22	GFP × tdTomato	Group 3 innate lymphoid cells	Intestinal inflammation	Changes in cell movement	Jarade et al. [25]
Eo	tdTomato, GFP	Eosinophils	Helminth infection	Cell recruitment/accumulation	Nguyen et al. [26]

*DSS* dextran sulfate sodium, *LPS* lipopolysaccharide

## Intestinal immune responses in inflammation

### Enterocolitis/inflammatory bowel disease (IBD)

In recent years, various new findings have been obtained using the mouse enterocolitis model and IVM technique [27, 28]. Observations targeting various immune cells are also progressing. For example, using LysMeGFP + mice as a neutrophil-targeted study, Lammers et al. revealed slowing of eGFP + neutrophils in vessels and influx into small intestinal mucosal tissue via formyl peptide receptor 1 within 2 h after oral gavage of gliadin, the immunogenic component of gluten and trigger of celiac disease [17]. Using TPLSM, another group reported the activities of extracellular signal-regulated kinase and protein kinase A in neutrophils in inflamed intestines [29]. In an oxazolone-induced colitis model, IVM with fluorescence resonance energy transfer technology showed frequent Ca^2+^ signaling in B cells of cecal patches during the early phase of colitis, suggesting B cell differentiation into plasma cells [30]. In a monocyte-focused study, interruption of the fractalkine–CX3CR1 axis ameliorated colitis through regulation of intravascular monocyte behaviors on the venous endothelium of inflamed colons in oxazolone-induced colitis models [31]. In a salmonella infection model, IVM revealed that CX3CR1 + macrophages and CD103 + dendritic cells efficiently phagocytosed salmonella using intraepithelial dendrites [19, 20]. Various T cells exist in the intestinal tract, and research using IVM has been widely conducted. A cell dynamics study of Foxp3 + regulatory T cells and intraepithelial CD4 + T cells revealed their distinct, but complementary roles in suppressing intestinal inflammation [23]. Lok et al. used a CD2/IL-13 double reporter mouse to image group 2 innate lymphoid cells [24]. They showed increases in the IL-13 + ILC2 size and movement in Peyer’s patch after helminth infection, but shorter cellular contacts with T cells. Additionally, T cells facilitated the patrolling attributes of group 3 innate lymphoid cells under inflammatory conditions by producing the chemokine CCL25 [25]. Intravital imaging has also shown that GPR55-deficient intraepithelial lymphocytes migrate faster and interact more extensively with epithelial cells [21]. Imaging of the jejunal mucosa in lipopolysaccharide-treated TcrdEGFP mice showed that ɤδ intraepithelial lymphocytes maintain prolonged contact with shedding enterocytes [22]. Another study focused on eosinophils and established EoCre/tdTomato^+/−^ mice [26]. Eosinophils were observed by IVM to rapidly surround murine-specific helminth parasites that invaded the small intestines. To image mast cells in vivo, c-Kit-eGFP mice or Mcpt5-CreROSA26-EYFP double transgenic can be used [32], but they have not been widely used for intravital imaging during intestinal inflammation. Moreover, Koike et al. imaged intestinal microcirculation in a murine necrotizing enterocolitis model using TPLSM and demonstrated that remote ischemic conditioning in the early stages of disease progression counteracted the poor microcirculatory response to formula feeding and preserved the arteriole flow velocity, diameter, and flow volume [33, 34].

IBD is a general term for diseases that cause uncontrolled chronic inflammation in the intestinal mucosa and generally refers to ulcerative colitis and Crohn’s disease. The dextran sulfate sodium (DSS) colitis model has been widely used as an animal model of IBD. Intravital imaging of DSS-induced colitis using TPLSM and an organ-stabilizing system revealed an irregularity and disappearance of crypts, infiltration of immune cells, and increased rolling of white blood cells along the vascular wall [35]. Administration of 5A peptide decreased leukocyte–endothelium interactions [36]. Furthermore, real-time imaging of a bacterial translocation model showed that RFP-*Escherichia* *coli* translocated from the luminal side of the intestines into blood vessels. Administration of steroids ameliorated intravital three-dimensional dynamic pathological changes caused by DSS-induced colitis [37]. Our observations showed that the gut microbiota affects the tissue repair process in DSS-induced colitis by facilitating blood-derived monocyte conversion from classical CCR2^hi^CX3CR1^lo^ monocytes to alternative CCR2^lo^CX3CR1^hi^ monocytes [18]. Peritoneal GATA6 + macrophages were microbiome-independently recruited to the colon in a DSS colitis model and contributed to ameliorating intestinal inflammation [38].

### Intestinal ischemia–reperfusion injury (IRI)

Intestinal IRI is related to various clinical conditions, such as ischemic enteritis, abdominal surgeries, and organ transplantation, and reduces patient survival because of bacterial translocation, systemic inflammation, intestinal necrosis, and multiple organ failure. We observed real-time neutrophil recruitment during IRI in small intestines using LysM^egfp^ mice and TPLSM [5]. This method enabled real-time assessment of neutrophil recruitment and pathophysiological changes in the intestinal wall in vivo. Voisin et al. showed that blocking neutrophil elastase-dependent neutrophil extravasation may be an effective strategy to reduce the number and activation of neutrophils in IRI, but it may also inhibit the recruitment of tissue-healing immune cells including monocytes and M2 macrophages [39]. Systemic treatment of mice with sulforaphane, an isothiocyanate with anti-inflammatory characteristics, reduced platelet activation and blocked leukocyte adhesion, significantly reducing leukocyte rolling at 2 and 8 h after intestinal IRI [40]. Moreover, the transfer of sulforaphane-treated platelets significantly reduced rolling leukocytes during reperfusion.

### NETs

Neutrophil extracellular traps (NETs) are the formation and release of sticky web-like structures composed of decondensed chromatin filaments that are decorated with histones and neutrophil granule proteins [41]. NETs play a pivotal role in intestinal infection by helping neutrophils catch and kill pathogens. Moreover, excessive NET formation has proinflammatory characteristics and induces innate immune responses [42]. Although many IVM studies on NETs have focused on the liver, Tanaka et al. successfully visualized NETs in vivo in the postcapillary venules of the murine cecum using the lipopolysaccharide-induced sepsis model [43]. They also observed leukocytes with cytoplasmic vacuoles that adhered to the vascular endothelium in LPS-treated mice at the subcellular level, and some of them released NETs. As discussed below, it has also been shown that colon cancer induces NETs in the liver, thereby promoting circulating cancer cell adhesion and liver metastasis [44]. Thus, the relationship between intestinal inflammation and NETs is a very interesting research field, but research using IVM has not progressed thus far and future development is desired.

## Immunity in colorectal cancer

IVM techniques have been applied to gastrointestinal cancer research. Mainly by analyzing colorectal cancer (CRC) metastasis models using tumor-specific transgenic mice and fluorescence-labeled cancer cell lines, the dynamics of tumor cells, tumor angiogenesis, chemotherapy responses in the liver microenvironment, and interactions with various immune cells have been clarified [45–48]. IVM through an abdominal imaging window allowed imaging a single step of CRC metastasis formation in the liver over 2 weeks [49]. Single extravasated CRC tumor cells proliferated to form pre-micrometastases, where tumor cells were active and motile within a confined region of the growing clone. Conversely, tumor cells within micrometastases were immotile. Fumagalli et al. reported real-time migration patterns of Lgr5 + and Lgr5 − CRC cells using a CRC mouse model generated by orthotopic transplantation of CRC organoids (RFP-Confetti and CRC Lgr5^eGFR^) [50]. They found that the majority of CRC cells in circulation were Lgr5 − and caused distant metastases in which Lgr5 + CRCs appeared. Another study focused on the cell cycle and performed intravital imaging of fluorescence ubiquitination-based cell cycle indicator (Fucci)-bearing human CRC cells [51]. Unexpectedly, S/G2/M Fucci green cells were more motile and invasive compared with Fucci red G1 cells. Intravital imaging of subcutaneously implanted CRC organoids using an imaging window enabled live genetic lineage tracing at the single-cell level over 30 days [52]. Dormant LGR5 + CRC stem cells are characterized by p27 expression, and IVM revealed that LGR5 + p27 + cells survive chemotherapy and then undergo clonal expansion. Intrasplenic injection of the CRC cell line MC38-RFP showed a significant increase in hepatic sinusoidal adhesion of MC38-RFP cells in tumor-bearing mice (TBM) compared to non-TBM, DAase1- or NET inhibitor-treated TBM, and *PAD4 − / − *TBM [44]. These data suggest that colon cancer induces NETs in the liver to facilitate the adhesion of circulating cancer cells and hepatic metastases. Similarly, the same group also reported high expression of NOD1 in human and murine CRC cell lines, and NOD1 activation augmented CRC cell adhesion in hepatic sinusoids [53]. As another model, Shimura et al. established a xenograft model of metastatic gastric cancer in the peritoneum using RFP-expressing gastric cancer cell line NUGC4 [54]. Overall, these results suggest that combining IVM data, which focuses on the real-time movement of cancer cells, and in vitro experimental results would enable the development of new treatment strategies for gastrointestinal cancer.

Interestingly, a recent study showed that peritoneal GATA6 + macrophages invade CRC liver metastases directly from the peritoneal cavity by sensing tumor-induced mesothelial injury [55]. Moreover, intravital imaging has revealed that peritoneal GATA6 + macrophages upregulate PD-L1 upon taking up apoptotic bodies from tumor cells and promote the growth of CRC liver metastases. These findings might lead to novel therapeutic strategies for CRC liver metastasis and its recurrence by manipulating peritoneal GATA6 + macrophages and considering the intraperitoneal cavity as a more effective route of drug administration.

## Mechanisms of tissue repair in intestinal injury

**Fig. 4:**
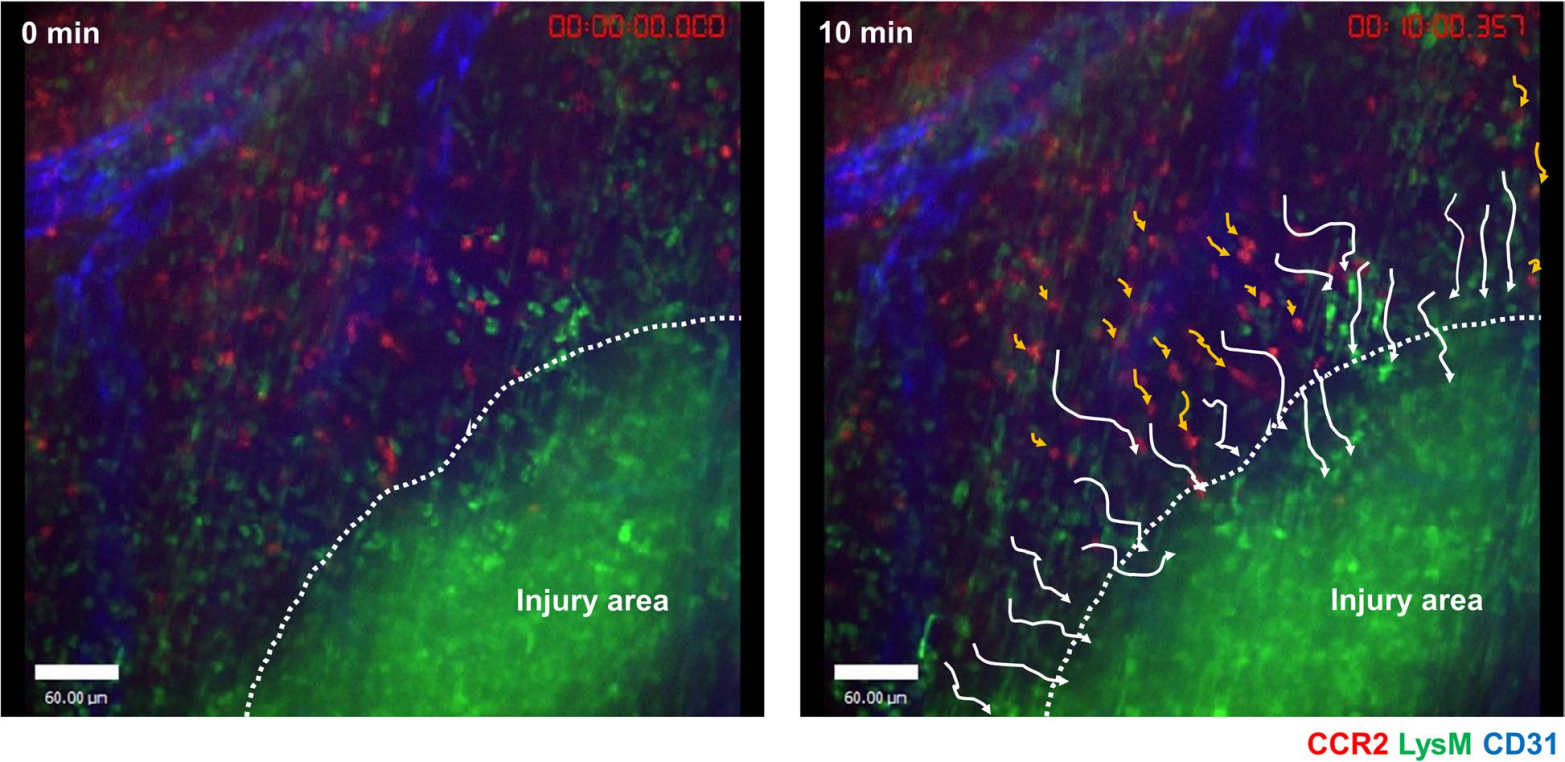
LysM^hi^ neutrophils and CCR2^+^ monocytes accumulate in injured intestines. Representative images of the colonic lamina propria over 10 min at 4 h after focal intestinal injury in LysM^GFP/+^CCR2^RFP/+^ mice. White and yellow arrows indicate migration paths of neutrophils and CCR2^+^ monocytes for 10 min, respectively

The study of the immune system during tissue damage is important for elucidating tissue repair mechanisms, and research using intravital imaging has provided many insights. Studies using a liver sterile injury model have shown that the first immune cells mobilized during tissue remodeling are platelets and neutrophils and recently revealed GATA6-positive peritoneal cavity macrophages [56, 57]. Subsequently, accumulation of CCR2-positive classical monocytes occurs, which are converted to CX3CR1-positive monocytes and macrophages that act in tissue repair [58]. iNKT cells orchestrate monocyte conversion from inflammation to resolution by producing interleukin-4 [59]. In a model of acute intestinal injury, IVM showed that neutrophils followed by CCR2-positive monocytes are accumulating into the injured area (Fig. 4). CCR2, Nr4a1, and the microbiome were necessary for appropriate monocyte recruitment, conversion, and development to mature CX3CR1-positive macrophages, allowing debris removal and rapid repair of the vasculature [18]. The same mechanisms are needed to repair colonic ulcers caused by DSS-induced colitis. Recently, we have shown that large F4/80^hi^GATA6-positive peritoneal cavity macrophages promptly accumulate at damaged intestinal sites via a direct route from the peritoneal cavity [38]. Compared with bloodstream-derived monocytes/macrophages, recruitment of cavity macrophages depended on ATP released by dead cells and exposed hyaluronan at the injury site. They contributed to the removal of necrotic cells, revascularization, and collagen deposition and thus resolution of intestinal tissue damage. The roles of platelets, neutrophils, and iNKT cells in intestinal tissue repair have not been investigated in detail and are a topic for further research.

The epithelial barrier function contributes to maintaining intestinal tissue homeostasis. Focusing on continuous renewal and turnover of the intestinal epithelium, imaging of intestinal crypts by IVM with an abdominal window and Lgr5^EGFP−Ires−CreERT2^ mice enabled tracing the fate of individual Lgr5 + intestinal stem cells and their progeny over time [60]. Bruens et al. used this approach and revealed that crypt fission and fusion in the intestinal epithelium regulate crypt numbers as a counterbalancing mechanism [61]. Another study showed that, when crypt cells were ablated, they were expelled from the crypt base by the rapid motion of crypt cells [62]. Subsequently, Lgr5 + stem cells and Paneth cells restored the alteration in the pattern. Interestingly, these mechanisms were impaired by inhibition of the ROCK pathway and aging. Using RAC1 knockout mice, it has also been shown that impairment of epithelial RAC1 functions causes cell overcrowding and epithelial leakage [63]. Future studies may reveal the mechanisms of the motion dynamics of crypt cells in more detail, leading to new insights into the intestinal epithelium repair process.

## Conclusions and future perspectives

The development of intravital imaging has provided new insights into in vivo, real-time immunological processes in many organs and disease models. We recently revealed that dysbiosis alters the localization of intestinal lamina propria macrophages [18], and imaging of the immuno-biome will be challenging, but very intriguing in the future. In vivo fluorescence imaging of the gastrointestinal system is a new research field and has a great advantage of being able to directly capture physiological phenomena in the living state. Because it is possible to elucidate the pathophysiology of intestinal inflammation, which was previously only evaluated statically, and to evaluate the mechanism-of-action of drugs over time, it is expected to be a new experimental model suitable for clinical research.

## Data Availability

Not applicable.

## Abbreviations

IVM: Intravital microscopy
TPLSM: Two-photon laser scanning microscopy
SHG: Second harmonic generation
IBD: Inflammatory bowel disease
DSS: Dextran sulfate sodium
IRI: Ischemia–reperfusion injury
NETs: Neutrophil extracellular traps
CRC: Colorectal cancer
